# 2-Cyano-5-({4-[*N*-methyl-*N*-(2-hy­droxy­eth­yl)amino] phen­yl}diazen­yl)pyridine

**DOI:** 10.1107/S1600536812041396

**Published:** 2012-10-06

**Authors:** Roberto Centore, Vincenzo Piccialli

**Affiliations:** a Dipartimento di Scienze Chimiche, Università degli Studi di Napoli ’Federico II’, Complesso di Monte S. Angelo, Via Cinthia, 80126 Napoli, Italy

## Abstract

In the title compound, C_15_H_15_N_5_O, the benzene and pyridine rings make a dihedral angle of 30.86 (7)°. In the crystal, chains of mol­ecules are wrapped around the screw axes into compressed helices, through hydrogen bonding between the hy­droxy and cyano groups. The chains are linked by weak C—H⋯N and C—H⋯O inter­actions. The π conjugated unit of the mol­ecule is almost perpendicular to the helix axis, and the formation of the helix is allowed by a *gauche*-type torsion angle in the hy­droxy­ethyl tail. In this way, consecutive chromophore units along the chain are placed in a strict anti­parallel arrangement, and this is energetically favoured because of the high dipole moment of the mol­ecule.

## Related literature

For general information on non-linear optical compounds, see: Singer *et al.* (1989 ▶); Dalton (2002 ▶). For structural and theoretical analysis of conjugation in push–pull mol­ecules, see: Gainsford *et al.* (2008 ▶); Centore *et al.* (2009 ▶); Capobianco *et al.* (2012 ▶). For the local packing modes of non-linear optical chromophores, see: Coe *et al.* (2000 ▶); Thallapally *et al.* (2002 ▶); Centore *et al.* (2006 ▶). For theoretical computations on similar compounds, see: Willets *et al.* (1992 ▶); Castaldo *et al.* (2002 ▶); Locatelli *et al.* (2005 ▶). For the CSD see: Allen (2002 ▶). For the synthesis of related compounds, see: Bruno *et al.* (2002 ▶); Centore *et al.* (2007 ▶); Centore *et al.* (2012 ▶).
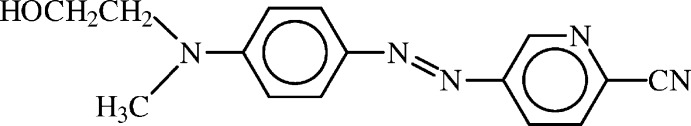


## Experimental

### 

#### Crystal data

C_15_H_15_N_5_O
*M* *_r_* = 281.32
Monoclinic, 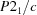

*a* = 17.755 (8) Å
*b* = 7.240 (4) Å
*c* = 11.045 (8) Å
β = 101.07 (5)°
*V* = 1393.4 (14) Å^3^
*Z* = 4
Mo *K*α radiation
μ = 0.09 mm^−1^
*T* = 293 K
0.40 × 0.10 × 0.05 mm

#### Data collection

Bruker–Nonius KappaCCD diffractometer
Absorption correction: multi-scan (*SADABS*; Bruker, 2001 ▶) *T*_min_ = 0.965, *T*_max_ = 0.996
8528 measured reflections
2403 independent reflections
1336 reflections with *I* > 2σ(*I*)
*R*_int_ = 0.069

#### Refinement

*R*[*F* ^2^ > 2σ(*F* ^2^)] = 0.052
*wR*(*F* ^2^) = 0.139
*S* = 1.02
2403 reflections
191 parameters
H-atom parameters constrained
Δρ_max_ = 0.14 e Å^−3^
Δρ_min_ = −0.21 e Å^−3^

### 

Data collection: *COLLECT* (Nonius, 1999 ▶); cell refinement: *CELLFITW* (Centore, 2004 ▶); data reduction: *EVALCCD* (Duisenberg *et al.*, 2003 ▶); program(s) used to solve structure: *SIR97* (Altomare *et al.*, 1999 ▶); program(s) used to refine structure: *SHELXL97* (Sheldrick, 2008 ▶); molecular graphics: *ORTEP-3 for Windows* (Farrugia, 2012 ▶) and *Mercury* (Macrae *et al.*, 2006 ▶); software used to prepare material for publication: *WinGX* (Farrugia, 2012 ▶).

## Supplementary Material

Crystal structure: contains datablock(s) global, I. DOI: 10.1107/S1600536812041396/fj2599sup1.cif

Structure factors: contains datablock(s) I. DOI: 10.1107/S1600536812041396/fj2599Isup2.hkl

Supplementary material file. DOI: 10.1107/S1600536812041396/fj2599Isup3.cml

Additional supplementary materials:  crystallographic information; 3D view; checkCIF report

## Figures and Tables

**Table 1:** Hydrogen-bond geometry (Å, °)

*D*—H⋯*A*	*D*—H	H⋯*A*	*D*⋯*A*	*D*—H⋯*A*
O1—H1*O*⋯N5^i^	1.03	1.92	2.925 (4)	165
C6—H6⋯N4^ii^	0.93	2.74	3.637 (4)	162
C2—H2*B*⋯O1^iii^	0.97	2.68	3.369 (4)	129

Symmetry codes: (i) 
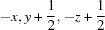
; (ii) 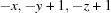; (iii) 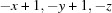.

**Fig. 1.**
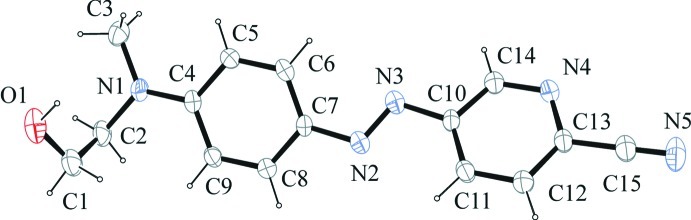
ORTEP view of the molecular structure of (I). Thermal ellipsoids are drawn at 30% probability level.

**Fig. 2.**
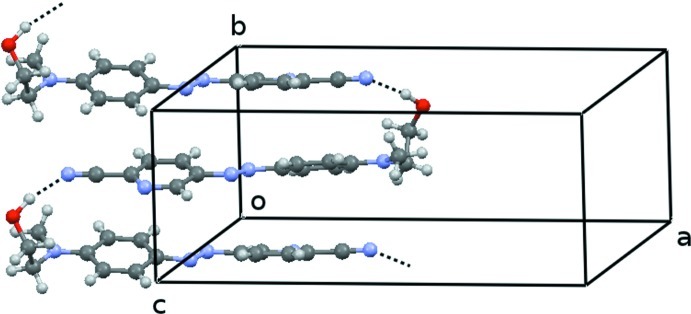
Row of H-bonded molecules of (I).

**Fig. 3.**
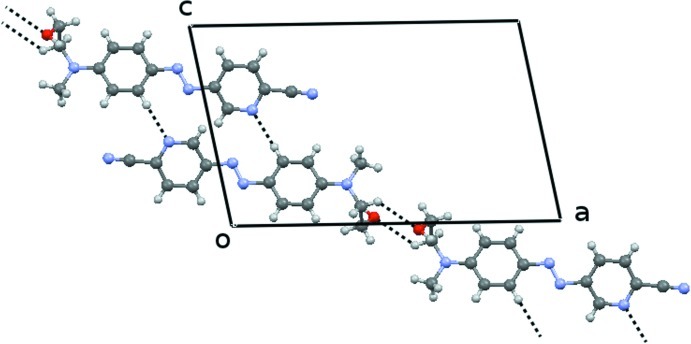
Lateral packing of (I) along b.

## supplementary crystallographic information

### Comment

Organic nonlinear optical (NLO) chromophores are currently under investigation because of possible applications in optical data processing (Dalton, 2002). The chemical investigation is mainly directed to the synthesis of chromophores of increasing quadratic nonlinear optical activity. However, also the structural investigation of NLO chromophores is relevant, pointing towards the quantitative evaluation of the structural parameters related to the conjugation in push-pull molecules (Gainsford *et al.*, 2008; Capobianco *et al.*, 2012) and to the rationalization of the local packing modes of chromophore units (Coe *et al.*, 2000; Thallapally *et al.*, 2002; Centore *et al.*, 2006).

Compound (I), 2-cyano-5-[4-(*N*-methyl-*N*-(2-hydroxyethyl)amino)- 1-diazenylphenyl]pyridine, is a typical push-pull azo-dye, containing the dialkylamino as donor group and the cyano as acceptor. Moreover, the cyano group is attached to an electron poor pyridine ring, and this should increase the electron withdrawing character. This compound has been used in the synthesis of cross-linked systems showing both piezoelectric and quadratic NLO behaviour (Centore *et al.*, 2012).

The molecular structure of (I) is shown in Fig. 1. The geometry around the donor N1 atom is substantially planar indicating *sp*^2^ hybridization (the sum of valence angles at N1 is 360°) and the pattern of bond lenghts within the adjacent phenyl ring shows a certain degree of quinoidal character. All these structural features are in accordance with the expected π conjugation and push-pull character of the chromophore group.

The two aromatic rings are not coplanar, the dihedral angle between the mean planes being 30.86 (7)°; this twist, which is mainly due to a torsion around the bond N3—C10, is not expected to negatively affect the quadratic NLO performances of the molecule, as it has been proved both theoretically and experimentally for similar chromophores (Castaldo *et al.*, 2002; Locatelli *et al.*, 2005).

Nonlinear optical properties of (I) have been determined by means of electro-optical absorption spectroscopy measurements in dioxane solution (Centore *et al.*, 2009). Relevant data are given in the Experimental part. We note that the dipole moment of the ground state is rather high and also the change in dipole moment between the ground and the first excited state is high, coherently with the expected charge-transfer character of the HOMO-LUMO transition. The quadratic NLO activity of (I), measured by the µβ_0_ product, is also significant, if we consider the simple chemical structure of the compound, and is comparable with the NLO reference compound DANS (Singer *et al.*, 1989).

Molecules in the crystal form rows through hydrogen bonds between hydroxy and cyano groups of consecutive molecules, Fig. 2 and Table 1. The chains, which have graph set symbol C^1^_1_(17), are wrapped around crystallographic binary screw axes. Actually, the pitch of the helix (*b*=7.240 (4) Å) is very short, as compared with the length of the molecule (N5···O1= 14.818 (8) Å), so the helix is very compressed; in fact, the π conjugated unit of the molecule is almost perpendicular to the helix axis, and the formation of the helix is is allowed by a *gauche*-type torsion angle in the hydroxyethyl tail. In this way, consecutive chromophore units along the chain are placed in a strict antiparallel arrangement, and this is energetically favoured because of the high dipole moment of the molecule.

Along (b+c) and a+b directions, the chains are held by weaker interactions involving C—H aromatic donor and pyridine N acceptor groups or C—H aliphatic donor and O acceptor groups (Fig. 3 and Table 1). Two dimeric ring patterns can be recognized, having graph set symbols *R*^2^_2_(16) and *R*^2^_2_(8); both are formed across crystallographic inversion centers.

A search within CSD (version 5.33) (Allen, 2002) has shown that these patterns are unprecedented in compounds containing *N*-methyl-*N*-2- hydroxyethylamino and azopyridyl-benzene mojeties.

### Experimental

(I) was prepared by diazotization of 5-amino-2-cyanopyridine followed by coupling with *N*-methyl-*N*-(2-hydroxyethyl)aniline. The procedure of diazo-coupling is analogous to that we have already described for the synthesis of similar diazo-chromophores (Bruno *et al.*, 2002; Centore *et al.*, 2007; Centore *et al.*, 2012). Purification of (I) was obtained by recrystallization from ethanol. The final yield for the diazotization/coupling step was 69%. Mp. 166 °C. Single crystals were obtained by slow evaporation from ethanol solutions. ^1^H-NMR (py-d_5_) δ 3.06 (3, 3H), 3.71 (t, 2H), 4.00 (t, 2H), 4.80 (s, 1H), 6.92 (d, 2H, J = 11 Hz), 7.84–8.19 (m, 4H), 9.26 (d, 1H, J = 2.6 Hz). Electro-optical absorption spectroscopy data of (I) (dioxane solution; quadratic hyperpolarizability is given according to the phenomenological convention (*X* convention) (Willets *et al.*, 1992)): λ_max_ = 461.3 nm, µ_g_ = 8.16 D, Δµ = 12.5 D, β_0_ = 69×10^-30^ esu, µ_g_β_0_ = 566×10^-48^ esu.

### Refinement

The H atom of the hydroxy group was located in difmap. All other H atoms were generated stereochemically. All H atoms were refined by the riding model with *U*_iso_=1.2×*U*_eq_ of the carrier atom (1.5 for H atoms of the methyl group).

### Crystal data

**Table:**

C_15_H_15_N_5_O	*D*_x_ = 1.341 Mg m^−^^3^
*M**_r_* = 281.32	Melting point: 439 K
Monoclinic, *P*2_1_/*c*	Mo *K*α radiation, λ = 0.71073 Å
*a* = 17.755 (8) Å	Cell parameters from 76 reflections
*b* = 7.240 (4) Å	θ = 5.6–23.2°
*c* = 11.045 (8) Å	µ = 0.09 mm^−^^1^
β = 101.07 (5)°	*T* = 293 K
*V* = 1393.4 (14) Å^3^	Plate, red
*Z* = 4	0.40 × 0.10 × 0.05 mm
*F*(000) = 592	

### Data collection

**Table:**

Bruker–Nonius KappaCCD diffractometer	2403 independent reflections
Radiation source: fine-focus sealed tube	1336 reflections with *I* > 2σ(*I*)
Graphite monochromator	*R*_int_ = 0.069
Detector resolution: 9 pixels mm^-1^	θ_max_ = 25.0°, θ_min_ = 3.1°
CCD rotation images, thick slices scans	*h* = −20→21
Absorption correction: multi-scan (*SADABS*; Bruker, 2001)	*k* = −7→8
*T*_min_ = 0.965, *T*_max_ = 0.996	*l* = −13→11
8528 measured reflections	

### Refinement

**Table:**

Refinement on *F*^2^	Primary atom site location: structure-invariant direct methods
Least-squares matrix: full	Secondary atom site location: difference Fourier map
*R*[*F*^2^ > 2σ(*F*^2^)] = 0.052	Hydrogen site location: inferred from neighbouring sites
*wR*(*F*^2^) = 0.139	H-atom parameters constrained
*S* = 1.02	*w* = 1/[σ^2^(*F*_o_^2^) + (0.0646*P*)^2^ + 0.0464*P*] where *P* = (*F*_o_^2^ + 2*F*_c_^2^)/3
2403 reflections	(Δ/σ)_max_ < 0.001
191 parameters	Δρ_max_ = 0.14 e Å^−^^3^
0 restraints	Δρ_min_ = −0.21 e Å^−^^3^

### Special details

**Table:**

Geometry. All e.s.d.'s (except the e.s.d. in the dihedral angle between two l.s. planes) are estimated using the full covariance matrix. The cell e.s.d.'s are taken into account individually in the estimation of e.s.d.'s in distances, angles and torsion angles; correlations between e.s.d.'s in cell parameters are only used when they are defined by crystal symmetry. An approximate (isotropic) treatment of cell e.s.d.'s is used for estimating e.s.d.'s involving l.s. planes.
Refinement. Refinement of *F*^2^ against ALL reflections. The weighted *R*-factor *wR* and goodness of fit *S* are based on *F*^2^, conventional *R*-factors *R* are based on *F*, with *F* set to zero for negative *F*^2^. The threshold expression of *F*^2^ > σ(*F*^2^) is used only for calculating *R*-factors(gt) *etc*. and is not relevant to the choice of reflections for refinement. *R*-factors based on *F*^2^ are statistically about twice as large as those based on *F*, and *R*- factors based on ALL data will be even larger.

### Fractional atomic coordinates and isotropic or equivalent isotropic displacement parameters (Å^2^)

**Table:**

	*x*	*y*	*z*	*U*_iso_*/*U*_eq_	
C1	0.39381 (17)	0.5114 (4)	−0.0089 (3)	0.0658 (9)	
H1A	0.4101	0.4652	−0.0821	0.079*	
H1B	0.3398	0.5431	−0.0314	0.079*	
C2	0.40363 (16)	0.3604 (3)	0.0877 (3)	0.0539 (7)	
H2A	0.3763	0.2511	0.0523	0.065*	
H2B	0.4576	0.3290	0.1102	0.065*	
C3	0.43153 (16)	0.4817 (4)	0.3024 (3)	0.0634 (8)	
H3A	0.4355	0.3973	0.3704	0.095*	
H3B	0.4154	0.6004	0.3266	0.095*	
H3C	0.4806	0.4931	0.2788	0.095*	
C4	0.29960 (14)	0.4002 (3)	0.2038 (2)	0.0389 (6)	
C5	0.27273 (14)	0.4488 (3)	0.3117 (2)	0.0426 (7)	
H5	0.3073	0.4906	0.3803	0.051*	
C6	0.19600 (15)	0.4353 (3)	0.3169 (2)	0.0416 (6)	
H6	0.1799	0.4664	0.3895	0.050*	
C7	0.14206 (14)	0.3759 (3)	0.2154 (2)	0.0356 (6)	
C8	0.16750 (14)	0.3312 (3)	0.1079 (2)	0.0412 (6)	
H8	0.1322	0.2941	0.0388	0.049*	
C9	0.24378 (14)	0.3407 (3)	0.1019 (2)	0.0423 (7)	
H9	0.2593	0.3072	0.0292	0.051*	
C10	−0.03818 (14)	0.3687 (3)	0.3055 (2)	0.0377 (6)	
C11	−0.09206 (15)	0.4028 (3)	0.2005 (2)	0.0445 (7)	
H11	−0.0770	0.4255	0.1258	0.053*	
C12	−0.16836 (15)	0.4026 (3)	0.2081 (2)	0.0479 (7)	
H12	−0.2061	0.4250	0.1387	0.058*	
C13	−0.18771 (15)	0.3687 (3)	0.3206 (3)	0.0420 (6)	
C14	−0.06318 (15)	0.3353 (3)	0.4146 (2)	0.0484 (7)	
H14	−0.0264	0.3124	0.4852	0.058*	
C15	−0.26695 (18)	0.3730 (3)	0.3352 (3)	0.0519 (7)	
N1	0.37562 (12)	0.4124 (3)	0.1987 (2)	0.0459 (6)	
N2	0.06251 (12)	0.3612 (2)	0.21080 (19)	0.0417 (5)	
N3	0.04319 (12)	0.3723 (3)	0.3147 (2)	0.0455 (6)	
N4	−0.13639 (13)	0.3339 (3)	0.4246 (2)	0.0493 (6)	
N5	−0.32935 (16)	0.3783 (3)	0.3473 (3)	0.0743 (8)	
O1	0.43618 (12)	0.6724 (3)	0.0323 (2)	0.0771 (7)	
H1O	0.4068	0.7588	0.0816	0.093*	

### Atomic displacement parameters (Å^2^)

**Table:**

	*U*^11^	*U*^22^	*U*^33^	*U*^12^	*U*^13^	*U*^23^
C1	0.0473 (19)	0.093 (2)	0.063 (2)	0.0003 (17)	0.0247 (16)	0.0034 (17)
C2	0.0400 (17)	0.0608 (17)	0.067 (2)	0.0028 (13)	0.0272 (15)	−0.0057 (14)
C3	0.0399 (18)	0.086 (2)	0.064 (2)	−0.0063 (15)	0.0079 (16)	0.0043 (16)
C4	0.0361 (17)	0.0363 (14)	0.0476 (17)	0.0008 (11)	0.0161 (13)	0.0059 (11)
C5	0.0417 (17)	0.0504 (15)	0.0365 (16)	−0.0018 (11)	0.0096 (13)	0.0015 (11)
C6	0.0458 (18)	0.0425 (14)	0.0411 (16)	0.0033 (11)	0.0202 (14)	0.0034 (11)
C7	0.0308 (15)	0.0362 (13)	0.0420 (16)	−0.0004 (10)	0.0128 (13)	0.0053 (11)
C8	0.0390 (17)	0.0465 (15)	0.0391 (16)	−0.0018 (11)	0.0102 (13)	−0.0007 (11)
C9	0.0440 (18)	0.0458 (15)	0.0411 (17)	−0.0047 (12)	0.0186 (13)	−0.0054 (11)
C10	0.0328 (16)	0.0368 (14)	0.0464 (17)	−0.0024 (10)	0.0152 (13)	−0.0026 (11)
C11	0.0447 (18)	0.0501 (15)	0.0432 (17)	−0.0018 (12)	0.0195 (14)	−0.0005 (11)
C12	0.0415 (19)	0.0572 (17)	0.0463 (18)	0.0016 (12)	0.0113 (14)	0.0006 (12)
C13	0.0404 (17)	0.0366 (14)	0.0537 (18)	−0.0061 (11)	0.0207 (15)	−0.0089 (12)
C14	0.0409 (19)	0.0608 (17)	0.0449 (18)	−0.0036 (13)	0.0117 (14)	0.0022 (12)
C15	0.049 (2)	0.0473 (16)	0.064 (2)	−0.0056 (13)	0.0236 (16)	−0.0076 (13)
N1	0.0332 (14)	0.0546 (13)	0.0525 (15)	0.0002 (10)	0.0153 (11)	−0.0010 (10)
N2	0.0466 (16)	0.0383 (12)	0.0435 (14)	0.0014 (9)	0.0174 (11)	0.0022 (9)
N3	0.0462 (16)	0.0468 (12)	0.0469 (14)	−0.0011 (10)	0.0180 (11)	0.0027 (10)
N4	0.0442 (16)	0.0613 (14)	0.0461 (15)	−0.0063 (11)	0.0183 (12)	−0.0022 (10)
N5	0.0514 (19)	0.0733 (17)	0.108 (2)	−0.0089 (13)	0.0397 (16)	−0.0089 (15)
O1	0.0668 (16)	0.0722 (14)	0.1043 (18)	−0.0004 (11)	0.0466 (13)	0.0101 (12)

### Geometric parameters (Å, º)

**Table:**

C1—O1	1.414 (3)	C7—N2	1.408 (3)
C1—C2	1.514 (4)	C8—C9	1.370 (3)
C1—H1A	0.9700	C8—H8	0.9300
C1—H1B	0.9700	C9—H9	0.9300
C2—N1	1.458 (3)	C10—C11	1.376 (4)
C2—H2A	0.9700	C10—C14	1.383 (3)
C2—H2B	0.9700	C10—N3	1.429 (3)
C3—N1	1.454 (3)	C11—C12	1.373 (3)
C3—H3A	0.9600	C11—H11	0.9300
C3—H3B	0.9600	C12—C13	1.373 (3)
C3—H3C	0.9600	C12—H12	0.9300
C4—N1	1.365 (3)	C13—N4	1.346 (3)
C4—C5	1.410 (3)	C13—C15	1.447 (4)
C4—C9	1.416 (3)	C14—N4	1.325 (3)
C5—C6	1.378 (3)	C14—H14	0.9300
C5—H5	0.9300	C15—N5	1.142 (3)
C6—C7	1.395 (3)	N2—N3	1.262 (3)
C6—H6	0.9300	O1—H1O	1.0341
C7—C8	1.387 (3)		
			
O1—C1—C2	112.8 (2)	C9—C8—C7	121.1 (2)
O1—C1—H1A	109.0	C9—C8—H8	119.4
C2—C1—H1A	109.0	C7—C8—H8	119.4
O1—C1—H1B	109.0	C8—C9—C4	121.6 (2)
C2—C1—H1B	109.0	C8—C9—H9	119.2
H1A—C1—H1B	107.8	C4—C9—H9	119.2
N1—C2—C1	113.2 (2)	C11—C10—C14	118.5 (2)
N1—C2—H2A	108.9	C11—C10—N3	125.9 (2)
C1—C2—H2A	108.9	C14—C10—N3	115.5 (2)
N1—C2—H2B	108.9	C12—C11—C10	118.9 (3)
C1—C2—H2B	108.9	C12—C11—H11	120.6
H2A—C2—H2B	107.8	C10—C11—H11	120.6
N1—C3—H3A	109.5	C13—C12—C11	118.5 (3)
N1—C3—H3B	109.5	C13—C12—H12	120.8
H3A—C3—H3B	109.5	C11—C12—H12	120.8
N1—C3—H3C	109.5	N4—C13—C12	124.0 (3)
H3A—C3—H3C	109.5	N4—C13—C15	115.0 (2)
H3B—C3—H3C	109.5	C12—C13—C15	121.0 (3)
N1—C4—C5	121.1 (2)	N4—C14—C10	123.9 (3)
N1—C4—C9	122.2 (2)	N4—C14—H14	118.1
C5—C4—C9	116.6 (2)	C10—C14—H14	118.1
C6—C5—C4	121.0 (2)	N5—C15—C13	179.3 (3)
C6—C5—H5	119.5	C4—N1—C3	121.4 (2)
C4—C5—H5	119.5	C4—N1—C2	121.2 (2)
C5—C6—C7	121.3 (2)	C3—N1—C2	117.4 (2)
C5—C6—H6	119.3	N3—N2—C7	114.1 (2)
C7—C6—H6	119.3	N2—N3—C10	112.4 (2)
C8—C7—C6	118.3 (2)	C14—N4—C13	116.2 (2)
C8—C7—N2	116.1 (2)	C1—O1—H1O	112.1
C6—C7—N2	125.6 (2)		
			
O1—C1—C2—N1	62.7 (3)	C11—C10—C14—N4	0.0 (3)
N1—C4—C5—C6	−179.6 (2)	N3—C10—C14—N4	177.4 (2)
C9—C4—C5—C6	1.1 (3)	C5—C4—N1—C3	−2.1 (3)
C4—C5—C6—C7	−1.0 (3)	C9—C4—N1—C3	177.2 (2)
C5—C6—C7—C8	−0.3 (3)	C5—C4—N1—C2	179.4 (2)
C5—C6—C7—N2	−178.6 (2)	C9—C4—N1—C2	−1.4 (3)
C6—C7—C8—C9	1.5 (3)	C1—C2—N1—C4	82.0 (3)
N2—C7—C8—C9	180.0 (2)	C1—C2—N1—C3	−96.6 (3)
C7—C8—C9—C4	−1.4 (3)	C8—C7—N2—N3	168.65 (18)
N1—C4—C9—C8	−179.2 (2)	C6—C7—N2—N3	−13.0 (3)
C5—C4—C9—C8	0.1 (3)	C7—N2—N3—C10	176.23 (17)
C14—C10—C11—C12	0.1 (3)	C11—C10—N3—N2	−18.0 (3)
N3—C10—C11—C12	−176.9 (2)	C14—C10—N3—N2	164.91 (19)
C10—C11—C12—C13	0.1 (3)	C10—C14—N4—C13	−0.4 (3)
C11—C12—C13—N4	−0.6 (4)	C12—C13—N4—C14	0.7 (3)
C11—C12—C13—C15	177.8 (2)	C15—C13—N4—C14	−177.8 (2)

### Hydrogen-bond geometry (Å, º)

**Table:**

*D*—H···*A*	*D*—H	H···*A*	*D*···*A*	*D*—H···*A*
O1—H1*O*···N5^i^	1.03	1.92	2.925 (4)	165
C6—H6···N4^ii^	0.93	2.74	3.637 (4)	162
C2—H2*B*···O1^iii^	0.97	2.68	3.369 (4)	129

Symmetry codes: (i) −*x*, *y*+1/2, −*z*+1/2; (ii) −*x*, −*y*+1, −*z*+1; (iii) −*x*+1, −*y*+1, −*z*.
